# The Roles of Vitamin D Levels, Gla-Rich Protein (GRP) and Matrix Gla Protein (MGP), and Inflammatory Markers in Predicting Mortality in Intensive Care Patients: A New Biomarker Link?

**DOI:** 10.3390/metabo14110620

**Published:** 2024-11-13

**Authors:** Fatih Seğmen, Semih Aydemir, Onur Küçük, Recep Dokuyucu

**Affiliations:** 1 Department of Intensive Care Unit, Ankara City Hospital, Ankara 06800, Turkey; drsegmen@gmail.com; 2 Department of Anesthesiology and Reanimation, Yenimahalle Training and Research Hospital, University of Yıldırım Beyazit, Ankara 06800, Turkey; drsemihaydemir@gmail.com; 3 Department of Anesthesiology and Reanimation, Ankara Atatürk Sanatorium Training and Research Hospital, University of Health Sciences, Ankara 06800, Turkey; dr.okucuk@gmail.com; 4 Department of Physiology, Medical Specialization Training Center (TUSMER), Ankara 06800, Turkey

**Keywords:** ICU mortality, neutrophil-to-lymphocyte ratio, platelet-to-lymphocyte ratio, pan-immune-inflammation value, Gla-rich protein, dephosphorylated uncarboxylated matrix Gla protein

## Abstract

Objectives: Identifying reliable biomarkers to predict mortality in critically ill patients is crucial for optimizing management in intensive care units (ICUs). Inflammatory and metabolic markers are increasingly recognized for their prognostic value. This study aims to evaluate the association of various inflammatory and metabolic markers with ICU mortality. Methods: This prospective observational study was conducted from January 2023 to January 2024 in the City Hospital’s ICU. A total of 160 critically ill patients were enrolled. Laboratory parameters, including white blood cell (WBC) count, red cell distribution width (RDW), platelet count, neutrophil count, mean platelet volume (MPV), monocyte count, lymphocyte count, procalcitonin (PCT), C-reactive protein (CRP), calcium (Ca^++^), and vitamin D levels, were analyzed. Additionally, ratios such as the platelet-to-lymphocyte ratio (PLR), neutrophil-to-lymphocyte ratio (NLR), systemic inflammatory index (SII), and pan-immune-inflammation value (PIV) were calculated. Plasma levels of Gla-rich protein (GRP) and dephosphorylated uncarboxylated matrix Gla protein (dp-ucMGP) were measured using ELISA. Results: The mean age of the patients included in the study was 60.5 ± 15.8 years. Cardiovascular disease was present in 72 patients (45%), respiratory system disease in 58 (36%), and chronic kidney disease (CKD) in 38 (24%). Additionally, 61 patients (38%) had diabetes, and 68 (42%) had hypertension. Inflammatory markers, including PLR, NLR, and PIV, were all significantly higher in non-survivors, while calcium and vitamin D levels were lower (*p* < 0.05). Higher WBC, RDW, neutrophil count, PLR, NLR, PIV, CRP, procalcitonin, GRP, and dp-ucMGP levels were positively correlated with longer hospital stays and increased mortality. In contrast, platelet and lymphocyte counts were negatively correlated with both outcomes (*p* < 0.05). Vitamin D levels showed an inverse relationship with both hospital stay and mortality, indicating that lower levels were associated with worse outcomes (*p* < 0.05). In multiple logistic regression analysis, elevated WBC count (OR = 1.20, *p* = 0.02), RDW (OR = 1.35, *p* = 0.01), neutrophil count (OR = 1.25, *p* = 0.01), MPV (OR = 1.20, *p* = 0.02), PLR (OR = 1.30, *p* = 0.01), NLR (OR = 1.40, *p* = 0.001), PIV (OR = 1.50, *p* = 0.001), CRP (OR = 1.32, *p* = 0.01), procalcitonin (OR = 1.45, *p* = 0.001), GRP (OR = 1.40, *p* = 0.001), and dp-ucMGP (OR = 1.30, *p* = 0.001) levels were significantly associated with increased mortality. Conclusions: Inflammatory and metabolic markers, particularly NLR, PLR, PIV, GRP, and dp-ucMGP, are strong predictors of mortality in ICU patients. These markers provide valuable insights for risk stratification and early identification of high-risk patients, potentially guiding more targeted interventions to improve outcomes.

## 1. Introduction

Patients admitted to the intensive care unit (ICU) are often in critical condition, with high mortality rates despite advancements in medical care. Early identification of those at the greatest risk of poor outcomes is essential for optimizing treatment strategies and improving survival [1]. In this context, biomarkers that reflect the body’s inflammatory and metabolic status have gained increasing attention for their ability to predict clinical outcomes in critically ill patients.

Inflammation plays a central role in the pathophysiology of many critical illnesses, including sepsis, acute respiratory distress syndrome (ARDS), and multi-organ failure [2,3]. The immune response in these conditions often becomes dysregulated, leading to an excessive inflammatory state that contributes to tissue damage and worsens prognosis [4]. As a result, inflammatory markers, such as the neutrophil-to-lymphocyte ratio (NLR) and platelet-to-lymphocyte ratio (PLR), have been studied extensively as simple, cost-effective indicators of systemic inflammation [5,6]. Previous research has shown that elevated NLR and PLR are associated with increased mortality in a variety of ICU populations, including patients with critical illnesses, cardiovascular disease, and respiratory failure [7,8,9,10].

While traditional inflammatory markers, such as C-reactive protein (CRP) and procalcitonin, remain widely used in ICU settings [11,12], newer composite markers, such as the pan-immune-inflammation value (PIV), offer a more comprehensive assessment of the patient’s inflammatory burden [13,14]. The PIV incorporates multiple inflammatory components, providing a broader view of the immune response. Studies have suggested that the PIV may outperform individual markers, such as CRP, in predicting mortality, making it a promising tool for risk stratification in critically ill patients [13,15,16].

In addition to inflammatory markers, metabolic indicators, such as Gla-rich protein (GRP) and dephosphorylated uncarboxylated matrix Gla protein (dp-ucMGP), have emerged as important predictors of ICU outcomes [17]. These markers are involved in vascular health and calcification processes, which are critical in patients with underlying cardiovascular or metabolic diseases [18]. GRP and dp-ucMGP have been linked to poor prognosis in conditions such as chronic kidney disease and diabetes, both of which are common in ICU populations [19]. Their role in predicting ICU mortality, however, has not been fully explored and warrants further investigation.

Vitamin D deficiency is another factor that has been associated with worse outcomes in ICU patients. Vitamin D is known for its role in regulating immune function and reducing inflammation. Several studies have identified low vitamin D levels as a risk factor for increased mortality in critically ill patients [20,21,22]. Despite this, the inclusion of vitamin D as a routine marker in ICU risk assessment remains debated, highlighting the need for further research on its prognostic value.

The aim of this study is to evaluate the predictive value of a range of inflammatory and metabolic markers in ICU patients, with a particular focus on identifying the markers most strongly associated with mortality. By integrating both traditional markers, such as CRP and procalcitonin, with novel indicators, such as PIV, GRP, and dp-ucMGP, we seek to improve risk stratification and provide a more comprehensive understanding of the underlying mechanisms contributing to mortality in critically ill patients.

## 2. Materials and Methods

### 2.1. Study Design and Study Population

This prospective, observational study was conducted at City Hospital between January 2023 and January 2024. Informed consent was obtained from all participants prior to inclusion. A total of 160 patients treated in the intensive care unit (ICU) were enrolled. The inclusion criteria were based on the admission guidelines set by the Ethics Committee of the Intensive Care Medicine Association [1]. Blood samples were collected immediately upon ICU admission, prior to the initiation of any treatments, to ensure that the baseline inflammatory and metabolic markers were not influenced by subsequent medical interventions.

A power analysis was conducted to ensure an adequate sample size for detecting significant associations between biomarkers and ICU outcomes, with a target power of 80% and a significance level of 0.05. Additionally, potential confounders, such as comorbidities and variations in treatment protocols, were adjusted for in the statistical analysis using multivariable regression models to enhance the validity of our findings.

Patients’ medical histories and ongoing treatments prior to ICU admission were documented. This included chronic medication use, such as antihypertensives, antidiabetics, anticoagulants, and any prior immunosuppressive therapy. The study specifically excluded patients who had received antioxidant therapy, as this could affect the inflammatory markers analyzed. Furthermore, pre-existing conditions and medication regimens were carefully reviewed to identify any factors that could influence the inflammatory or metabolic status, ensuring a thorough assessment of potential confounding variables.

### 2.2. Laboratory Parameters

Detailed laboratory parameters included the white blood cell (WBC) count, red cell distribution width (RDW), platelet count, neutrophil count, mean platelet volume (MPV), monocyte count, lymphocyte count, procalcitonin (PCT), C-reactive protein (CRP), serum calcium levels, and vitamin D levels. These were measured using standardized automated hematological analyzers and biochemical assays. Specific ratios, such as the platelet-to-lymphocyte ratio (PLR), neutrophil-to-lymphocyte ratio (NLR), and the systemic inflammatory index (SII), were calculated using established formulas. The pan-immune-inflammation value (PIV) was derived to provide a comprehensive measure of the inflammatory burden [2]. Patient outcomes, including the length of hospital stay and mortality rates during hospitalization, were recorded, and their relationship with laboratory parameters was analyzed. Plasma levels of Gla-rich protein (GRP) and dephosphorylated uncarboxylated matrix Gla protein (dp-ucMGP) were quantified using enzyme-linked immunosorbent assays (ELISAs) following the manufacturers’ guidelines, with all assays performed in duplicate to ensure accuracy.

### 2.3. Measurements of Gla-Rich Protein (GRP) and Dephosphorylated Uncarboxylated Matrix Gla Protein (dp-ucMGP)

Plasma levels of circulating dp-ucMGP and GRP were measured using commercial enzyme-linked immunosorbent assay (ELISA) kits (RayBiotech Inc., Peachtree Corners, GA, USA). dp-ucMGP was measured with a competitive mono-antibody assay (Cusabio, Wuhan, China), while GRP levels were determined using a separate ELISA kit (Develop, China). The ELISA procedure involved multiplying all results by a factor of 15 to account for sample dilution. Positive and negative controls were included: the positive control featured a known concentration of recombinant GRP and MGP, and the negative control utilized phosphate-buffered saline (PBS) to ensure the absence of nonspecific binding.

The minimum detectable concentrations for human GRP and MGP were 1.23 ng/mL and 50 pg/mL, respectively. These detection thresholds are critical for distinguishing between inflammatory and non-inflammatory states. GRP is considered a marker of vascular health and is implicated in inflammatory processes, with levels typically increasing in response to inflammation. Values above the minimum detectable concentration of 1.23 ng/mL indicate the presence of inflammation-related activity. Similarly, dp-ucMGP, which plays a role in inhibiting vascular calcification, can be influenced by inflammatory conditions, with higher levels suggesting pathological vascular responses. Values exceeding the 50 pg/mL limit indicate potential involvement in inflammatory or pathological processes [17].

To ensure accurate results, values exceeding the assay’s maximum detection limits were capped at 3000 ng/mL for GRP and 1200 ng/mL for MGP, following the ELISA kit’s protocols. This approach allows for the reliable assessment of inflammatory and non-inflammatory states based on GRP and dp-ucMGP levels, providing meaningful insights into the inflammatory status of ICU patients.

### 2.4. Statistical Analysis

Data analysis was conducted using SPSS software, version 27.0 (SPSS Inc., Chicago, IL, USA). Continuous variables were presented as mean values ± standard deviations, and categorical variables as proportions. The Kolmogorov–Smirnov test was applied to assess the normality of data distribution. Parametric tests were used for normally distributed data, while non-parametric methods were used for data that did not follow a normal distribution. The chi-square test was employed to analyze categorical variables, while the Student’s *t*-test was used to compare means between two independent groups. Pearson correlation coefficients were calculated to assess the strength and direction of relationships between continuous variables. A linear regression analysis was conducted to evaluate the effect of parameters on mortality. To ensure the reliability of our logistic regression model, we performed multicollinearity tests among the inflammatory markers (NLR, PLR, and PIV) using the variance inflation factor (VIF). All VIF values were below the commonly accepted threshold of 5, indicating that multicollinearity was not a significant concern in our analysis, thus strengthening the robustness of our findings. A *p*-value of <0.05 was considered statistically significant.

## 3. Results

Sociodemographic and laboratory findings of patients in the ICU are shown in Table 1. The study included 160 patients admitted to the ICU, with a mean age of 60.5 ± 15.8 years. Among the patients, 92 (58%) were male and 68 (42%) were female. Cardiovascular disease was present in 72 patients (45%), respiratory system disease in 58 (36%), and chronic kidney disease (CKD) in 38 (24%). Additionally, 61 patients (38%) had diabetes, and 68 (42%) had hypertension. The laboratory findings revealed a mean WBC count of 11.2 ± 3.8 × 10^3^/μL, RDW of 14.5 ± 2.1%, and a platelet count of 190 ± 58 × 10^3^/μL. Neutrophil counts were 7.5 ± 2.9 × 10^3^/μL, monocyte counts were 0.7 ± 0.3 × 10^3^/μL, and lymphocyte counts were 1.0 ± 0.4 × 10^3^/μL. The PLR was 180 ± 35, while the NLR was 7.1 ± 3.0. The PIV was 540 ± 110. Key biochemical markers included calcium levels at 8.9 ± 0.7 mg/dL, C-reactive protein (CRP) levels at 65 ± 22 mg/L, and procalcitonin levels at 2.8 ± 1.2 ng/L. The mean vitamin D level was 18.5 ± 6.2 ng/mL. GRP levels were 0.81 ± 0.6 μg/mL, and dp-ucMGP levels were 720 ± 225 pmol/L. The mean hospital stay was 15.3 ± 6.5 days. ICU mortality was observed in 25% of the patients, with 40 non-survivors and 120 survivors (75%; Table 1).

A comparison of parameters of survival and mortality of patients in the ICU is shown in Table 2. RDW was also elevated in non-survivors (15.2 ± 2.4%) compared to survivors (14.0 ± 1.8%; *p* = 0.03). Platelet counts were significantly lower in non-survivors (160 ± 50 × 10^3^/μL) compared to survivors (210 ± 60 × 10^3^/μL; *p* = 0.01). Neutrophil counts were higher in non-survivors (8.5 ± 3.0 × 10^3^/μL) compared to survivors (7.0 ± 2.5 × 10^3^/μL; *p* = 0.02), while monocyte counts were slightly elevated in non-survivors (0.9 ± 0.4 × 10^3^/μL) compared to survivors (0.7 ± 0.3 × 10^3^/μL; *p* = 0.05). Lymphocyte counts were significantly lower in the mortality group (0.9 ± 0.3 × 10^3^/μL) than in the survivor group (1.2 ± 0.4 × 10^3^/μL; *p* = 0.03). The PLR was significantly higher in non-survivors (230 ± 50) compared to survivors (175 ± 35; *p* = 0.02). Similarly, the NLR was elevated in the mortality group (9.0 ± 3.5) versus the survivor group (6.0 ± 2.5; *p* = 0.01). The PIV was significantly elevated in non-survivors (610 ± 120) compared to survivors (450 ± 110; *p* = 0.01). Calcium levels were lower in non-survivors (8.4 ± 0.7 mg/dL) compared to survivors (8.8 ± 0.6 mg/dL; *p* = 0.04), while CRP levels were significantly higher in non-survivors (78 ± 25 mg/L) than survivors (32 ± 10 mg/L; *p* = 0.01). Procalcitonin levels were also elevated in the mortality group (3.8 ± 1.5 ng/L) compared to the survivor group (2.5 ± 1.1 ng/L; *p* = 0.03). Vitamin D levels were significantly lower in non-survivors (12.5 ± 5.0 ng/mL) compared to survivors (19.0 ± 6.0 ng/mL; *p* = 0.02). GRP levels were higher in non-survivors (0.98 ± 0.6 μg/mL) compared to survivors (0.76 ± 0.5 μg/mL; *p* = 0.03), while dp-ucMGP levels were significantly elevated in non-survivors (920 ± 180 pmol/L) compared to survivors (650 ± 210 pmol/L; *p* = 0.02; Table 2).

The correlation between the length of the hospital stay and ICU mortality with various clinical and laboratory parameters is shown in Table 3. WBC count was positively correlated with both the hospital stay (r = 0.28, *p* = 0.02) and ICU mortality (r = 0.35, *p* = 0.01). RDW also showed a significant positive correlation with hospital stay (r = 0.30, *p* = 0.01) and ICU mortality (r = 0.40, *p* = 0.01). Platelet count was negatively correlated with both the hospital stay (r = −0.20, *p* = 0.04) and ICU mortality (r = −0.25, *p* = 0.03). Neutrophil count demonstrated a positive correlation with hospital stay (r = 0.22, *p* = 0.03) and ICU mortality (r = 0.30, *p* = 0.02). MPV was positively correlated with hospital stay (r = 0.18, *p* = 0.05) and ICU mortality (r = 0.25, *p* = 0.03). Lymphocyte count showed a significant negative correlation with both the hospital stay (r = −0.25, *p* = 0.03) and ICU mortality (r = −0.30, *p* = 0.02). The PLR was positively correlated with hospital stay (r = 0.32, *p* = 0.01) and ICU mortality (r = 0.35, *p* = 0.01), while the NLR exhibited the strongest correlation with both the hospital stay (r = 0.40, *p* = 0.01) and ICU mortality (r = 0.45, *p* = 0.01). The PIV also showed a significant positive correlation with both the hospital stay (r = 0.38, *p* = 0.01) and ICU mortality (r = 0.42, *p* = 0.01). Serum calcium levels were not significantly correlated with hospital stay (r = −0.15, *p* = 0.08) or ICU mortality (r = −0.18, *p* = 0.06), but CRP levels were positively correlated with both the hospital stay (r = 0.28, *p* = 0.02) and ICU mortality (r = 0.32, *p* = 0.01). Procalcitonin levels also showed significant positive correlations with hospital stay (r = 0.35, *p* = 0.01) and ICU mortality (r = 0.40, *p* = 0.01). Vitamin D levels were inversely correlated with both the hospital stay (r = −0.30, *p* = 0.02) and ICU mortality (r = −0.40, *p* = 0.01), suggesting that lower levels of vitamin D are associated with worse outcomes. Finally, GRP levels were positively correlated with both the hospital stay (r = 0.35, *p* = 0.01) and ICU mortality (r = 0.38, *p* = 0.01), while dp-ucMGP levels showed the strongest positive correlation with hospital stay (r = 0.40, *p* = 0.01) and ICU mortality (r = 0.50, *p* = 0.001; Table 3).

Linear regression analysis results for the mortality of patients in the ICU are shown in Table 4. WBC count was a significant predictor of mortality (B = 0.15, *p* = 0.01). RDW also showed a strong association with mortality (B = 0.30, *p* = 0.001). Platelet count was inversely associated with mortality (B = −0.10, beta = −0.25, *p* = 0.02), while neutrophil count showed a positive association (B = 0.18, beta = 0.22, *p* = 0.01). MPV was also significantly associated with increased mortality (B = 0.08, beta = 0.20, *p* = 0.02). Monocyte count (B = 0.12, beta = 0.15, *p* = 0.03) and lymphocyte count (B = −0.20, beta = −0.25, *p* = 0.01) were also significant predictors, indicating that higher monocyte levels and lower lymphocyte levels are associated with an increased mortality risk. Inflammatory markers, such as the PLR (B = 0.22, beta = 0.30, *p* = 0.01) and the NLR (B = 0.35, beta = 0.40, *p* = 0.001), were both significantly associated with increased mortality. The PIV demonstrated a strong correlation with mortality (B = 0.40, beta = 0.42, *p* = 0.001), reinforcing the importance of inflammation as a predictor of outcomes. Calcium levels (B = −0.10, beta = −0.18, *p* = 0.02) and CRP levels (B = 0.28, beta = 0.32, *p* = 0.01) also emerged as significant predictors. Procalcitonin levels were strongly associated with mortality (B = 0.35, beta = 0.40, *p* = 0.001). Vitamin D levels were inversely correlated with mortality (B = −0.40, beta = −0.45, *p* = 0.001), suggesting that lower vitamin D levels are linked to worse outcomes. GRP levels (B = 0.25, beta = 0.35, *p* = 0.01) and dp-ucMGP levels (B = 0.20, beta = 0.30, *p* = 0.01) were also found to be significant predictors of ICU mortality (Table 4).

Multiple logistic regression analysis results for the mortality of patients in the ICU are shown in Table 5. WBC count was significantly associated with ICU mortality, with an odds ratio (OR) of 1.20 (95% CI: 1.05–1.35, *p* = 0.02). RDW also emerged as a strong predictor of mortality (OR = 1.35, 95% CI: 1.10–1.60, *p* = 0.01). Platelet count was inversely associated with ICU mortality (OR = 0.85, 95% CI: 0.75–0.95, *p* = 0.02), while neutrophil count showed a significant positive association with mortality (OR = 1.25, 95% CI: 1.10–1.40, *p* = 0.01). MPV was also an independent predictor (OR = 1.20, 95% CI: 1.05–1.35, *p* = 0.02). Monocyte count approached significance (OR = 1.10, 95% CI: 0.95–1.25, *p* = 0.08), while lymphocyte count was inversely associated with mortality (OR = 0.85, 95% CI: 0.75–0.95, *p* = 0.03). Inflammatory markers were strong predictors of ICU mortality. The PLR was associated with increased mortality (OR = 1.30, 95% CI: 1.10–1.50, *p* = 0.01), as was the NLR (OR = 1.40, 95% CI: 1.20–1.60, *p* = 0.001). The PIV showed the strongest association with mortality (OR = 1.50, 95% CI: 1.30–1.70, *p* = 0.001). Calcium levels were marginally significant, with a protective effect (OR = 0.90, 95% CI: 0.80–1.00, *p* = 0.05), while CRP was positively associated with mortality (OR = 1.32, 95% CI: 1.15–1.50, *p* = 0.01). Procalcitonin levels were another strong predictor (OR = 1.45, 95% CI: 1.25–1.65, *p* = 0.001). Vitamin D levels were inversely associated with ICU mortality (OR = 0.30, 95% CI: 1.10–1.52, *p* = 0.001). GRP (OR = 1.40, 95% CI: 1.18–1.62, *p* = 0.001) and dp-ucMGP (OR = 1.30, 95% CI: 1.12–1.48, *p* = 0.001) were both significantly associated with increased ICU mortality (Table 5).

**Figure 1:**
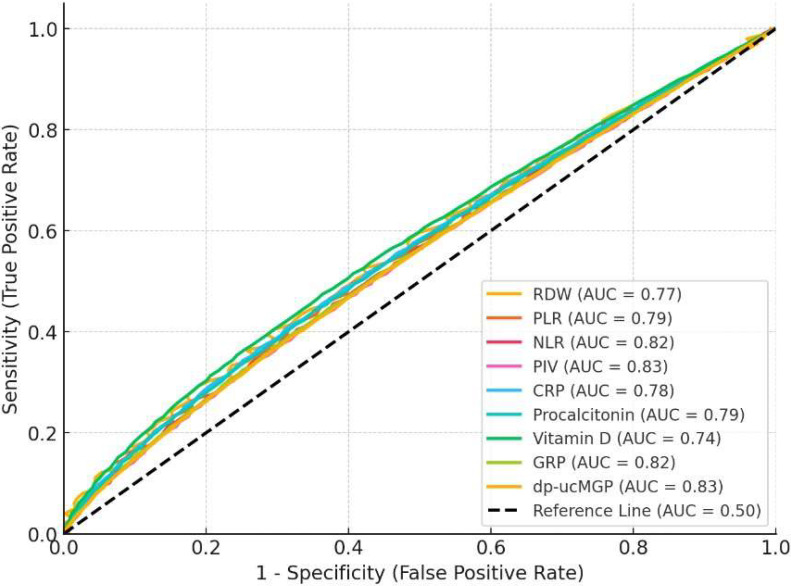
ROC analysis results in patients with mortality.

Receiver operating characteristic (ROC) analysis results in patients with mortality are shown in Table 6. RDW had a cut-off value of >14.8, with a sensitivity of 0.78, specificity of 0.72, and an AUC of 0.77 (95% CI: 0.70–0.83, *p* = 0.01). The PLR with a cut-off of >200 demonstrated a sensitivity of 0.81, specificity of 0.74, and an AUC of 0.79 (95% CI: 0.72–0.85, *p* = 0.001). The NLR with a cut-off of >7.5 exhibited a high predictive value, with a sensitivity of 0.84, specificity of 0.76, and an AUC of 0.82 (95% CI: 0.75–0.88, *p* = 0.001). Similarly, the PIV with a cut-off of >520 showed a sensitivity of 0.85, specificity of 0.79, and an AUC of 0.83 (95% CI: 0.77–0.89, *p* = 0.001). CRP levels > 40 mg/L had a sensitivity of 0.80, specificity of 0.75, and an AUC of 0.78 (95% CI: 0.72–0.84, *p* = 0.01), while procalcitonin levels > 3.0 ng/L demonstrated a sensitivity of 0.82, specificity of 0.74, and an AUC of 0.79 (95% CI: 0.73–0.86, *p* = 0.001). Vitamin D levels < 12 ng/mL had a sensitivity of 0.75, specificity of 0.72, and an AUC of 0.74 (95% CI: 0.68–0.80, *p* = 0.01). GRP levels > 0.90 μg/mL showed a sensitivity of 0.83, specificity of 0.77, and an AUC of 0.82 (95% CI: 0.75–0.88, *p* = 0.001). Finally, dp-ucMGP levels > 720 pmol/L exhibited the highest predictive value, with a sensitivity of 0.85, specificity of 0.80, and an AUC of 0.83 (95% CI: 0.76–0.89, *p* = 0.001; Table 6; Figure 1).

## 4. Discussion

**Figure 2:**
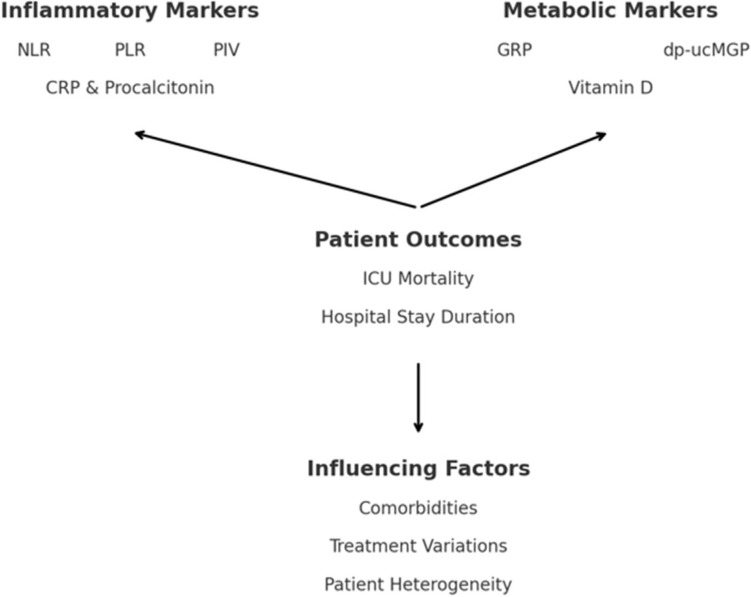
Conceptual scheme of biomarkers and ICU outcomes.

In this study, we identified several clinical and biochemical parameters that were significantly associated with ICU mortality. Among these, inflammatory markers, such as the neutrophil-to-lymphocyte ratio (NLR), platelet-to-lymphocyte ratio (PLR), and pan-immune-inflammation value (PIV), were strong predictors of poor outcomes. Furthermore, biomarkers, such as GRP and dp-ucMGP, which reflect vascular health and calcification processes, also emerged as independent predictors of ICU mortality (Figure 2).

The importance of NLR in predicting ICU mortality has been well documented across various studies. Moisa et al. reported that an elevated NLR was an independent predictor of mortality in critically ill patients, particularly those with COVID-19 [23]. Yang et al. found that higher NLR values were associated with significantly increased mortality in both sepsis and non-sepsis ICU patients [24]. Our study reinforced the utility of the NLR as a simple yet effective inflammatory marker that reflects immune imbalance in critical illness.

The PLR has also emerged as a significant predictor of mortality in ICU patients [9,25]. Zhai et al. showed the prognostic value of the PLR in ICU settings, particularly in patients with systemic inflammatory conditions. Furthermore, a study by Moosmann et al. showed that a high PLR was correlated with increased mortality in pediatric ICU patients, suggesting that the PLR can be a reliable marker across different patient populations and age groups [26]. In our study, the PLR was associated with increased mortality and, with a cut-off of >200, demonstrated a sensitivity of 0.81, specificity of 0.74, and an AUC of 0.79 for ICU mortality.

The predictive power of the PIV in our study is supported by recent evidence in critical care literature [27,28]. Gambichler et al. demonstrated the utility of the PIV in assessing the severity of inflammatory responses in COVID-19 patients, showing a strong correlation between an elevated PIV and increased mortality [29]. Similarly, Hafez et al. confirmed the prognostic value of the PIV in non-COVID ICU patients, particularly those with multi-organ failure [30]. In our study, the PIV levels were significantly higher in patients in the non-survivor group than in the survivor group. The PIV was positively correlated with both the length of hospital stay and ICU mortality, indicating that higher PIV levels were associated with longer hospital stays and an increased risk of death. In addition, the PIV with a cut-off of >520 showed a sensitivity of 0.85, specificity of 0.79, and an AUC of 0.83 for ICU mortality.

The association between GRP and ICU mortality in our study, the first in the literature, expands on previous research linking GRP to vascular calcification and cardiovascular outcomes [31,32,33]. Studies by Dahlberg et al. and Desai et al. showed that elevated GRP levels are associated with an increased risk of cardiovascular events and mortality in patients with chronic kidney disease and diabetes, conditions that are common among ICU patients [31,32]. Our study added further evidence that GRP could be a valuable marker in ICU settings, particularly in critically ill patients with underlying vascular complications. In addition, GRP levels > 0.90 μg/mL showed a sensitivity of 0.83, specificity of 0.77, and an AUC of 0.82 for ICU mortality.

Likewise, dp-ucMGP has been increasingly recognized as a marker of vascular health and calcification [34,35,36]. Roumeliotis et al. found that high levels of dp-ucMGP were associated with poor outcomes in patients on hemodialysis, suggesting that vascular calcification plays a key role in the pathophysiology of critical illness [37]. Willeit et al. also identified dp-ucMGP as a strong predictor of cardiovascular mortality in elderly patients, reinforcing the idea that dp-ucMGP could serve as an important prognostic marker across various high-risk patient populations, including those in the ICU [38]. In our study, it was found that dp-ucMGP levels were strongly associated with mortality in the ICU. In the non-survivor group, dp-ucMGP levels were significantly higher than in the survivor group. It was also observed that dp-ucMGP levels were positively correlated with both the length of hospital stay and ICU mortality, meaning that higher dp-ucMGP levels were associated with a longer hospital stay and an increased risk of death. In addition, dp-ucMGP exhibited strong predictive performance for ICU mortality, with a cut-off value of >720 pmol/L, a sensitivity of 0.85, specificity of 0.80, and an area under the curve (AUC) of 0.83.

Vitamin D deficiency has long been associated with poor outcomes in critically ill patients, and our findings are consistent with previous studies [39,40]. Charoenngam et al. found that vitamin D levels below 12 ng/mL were strongly associated with increased mortality in adult patients with COVID-19 [39]. Cervero et al. demonstrated that ICU patients with vitamin D deficiency had a 1.5-fold higher risk of mortality compared to those with adequate vitamin D levels [40]. Vitamin D’s immunomodulatory and anti-inflammatory properties are well documented, and our results further emphasized its critical role in maintaining immune function and reducing the mortality risk in critically ill patients. Vitamin D levels were significantly lower in the non-survivor group (12.5 ± 5.0 ng/mL) compared to the survivor group (19.0 ± 6.0 ng/mL). Additionally, in correlation analyses, vitamin D levels were inversely associated with both the length of hospital stay and ICU mortality. In regression analysis, vitamin D levels were found to be a strong negative predictor of ICU mortality. Vitamin D levels < 12 ng/mL had a sensitivity of 0.75, specificity of 0.72, and an AUC of 0.74 for ICU mortality.

Although newer markers, such as the NLR, PLR, and PIV, are gaining prominence, traditional markers, such as CRP and procalcitonin, remain relevant in ICU prognostication. Harikrishna et al. stated that procalcitonin levels above 3 ng/mL were associated with significantly increased mortality in septic ICU patients [41]. Additionally, CRP, which has long been used as a marker of inflammation, was confirmed by a study from Gosav et al. to be a reliable predictor of adverse outcomes, particularly when combined with other inflammatory markers, such as NLR or PLR [42]. These studies support our findings that while CRP and procalcitonin are useful on their own, their predictive value is enhanced when integrated with more comprehensive inflammatory indices.

Multiple studies have evaluated the benefits of combining inflammatory markers for improved predictive accuracy. For example, Tian et al. showed that combining NLR, PLR, and procalcitonin significantly improved the prediction of mortality in septic patients compared to using any single marker [43]. Similarly, the work of Chen et al. on COVID-19 patients highlighted the value of integrating multiple inflammatory and metabolic markers, including vitamin D and CRP, to create a more robust prognostic model [44]. These studies, in line with our findings, demonstrated that a multifaceted approach combining traditional and novel markers can greatly enhance risk stratification in critically ill patients. In our study, the NLR, PLR, PIV, procalcitonin, GRP, dp-ucMGP, and vitamin D were particularly significant in predicting mortality in the ICU.

Our findings revealed that, in addition to inflammatory markers, such as the NLR, PLR, and PIV, other parameters, such as the red cell distribution width (RDW), were significant predictors of ICU mortality. Elevated RDW has been associated with worse outcomes due to its reflection of chronic inflammation, oxidative stress, and impaired erythropoiesis, all of which contribute to altered oxygen transport and systemic dysregulation in critically ill patients. Previous studies, such as those by Patel et al. and Zhong et al., have similarly identified RDW as an independent risk factor for mortality in ICU settings, highlighting its prognostic value [45,46]. Our results align with these findings, as we observed a strong correlation between higher RDW levels and an increased mortality risk. The underlying mechanisms may involve chronic endothelial dysfunction, impaired red blood cell deformability, and the inflammatory cascade, which exacerbates multi-organ failure. Discussing these mechanisms provides a more comprehensive understanding of the complex interplay between hematological and inflammatory factors in predicting patient outcomes. Future research should further investigate these associations to refine risk stratification in critically ill populations.

The correlation coefficients (R values) between certain parameters and ICU mortality or the duration of hospitalization were not as strong as expected. This may be due to several factors. First, the complexity of critically ill patients involves multiple overlapping pathophysiological processes, making it difficult for any single parameter to strongly predict outcomes. Inflammatory and metabolic responses in the ICU setting can be influenced by a wide array of factors, including underlying comorbidities, variations in treatment protocols, and the dynamic nature of critical illness. Additionally, the heterogeneity of the study population may have diluted the strength of these correlations, as patients presented with diverse primary diagnoses and severity levels. Furthermore, the multifactorial nature of mortality and prolonged hospital stays in the ICU suggests that a combination of biomarkers, rather than individual ones, may be more effective for prediction. Despite these limitations, this study still provided valuable insights into how these parameters contribute to risk stratification when considered collectively.

### Limitations of the Study

This single-center observational study’s findings may not be fully generalizable to other ICU populations. While associations between markers and mortality were observed, causal relationships remain unclear. A key limitation was the exclusion of pro-inflammatory cytokines, such as IL-1β and NLRP3, which are gold standards for distinguishing between general inflammation and infection. This omission limited the study’s ability to comprehensively interpret inflammatory responses. Additionally, the study did not address potential coagulopathy, which could significantly impact outcomes in critically ill patients and may influence the associations observed. Variations in treatment protocols may also have influenced the results. Despite these limitations, the study is notable for being among the first to explore these specific biomarkers in ICU mortality and for providing a comprehensive analysis that offers practical implications for clinical risk assessment.

## 5. Conclusions

This study underscored the significant predictive value of inflammatory markers, such as the NLR, PLR, and PIV, along with biochemical parameters, such as GRP and dp-ucMGP, in determining ICU mortality. These findings suggested that a combination of immune and metabolic markers can enhance risk stratification in critically ill patients, providing valuable insights for clinical decision-making. The integration of these biomarkers into routine assessments may allow for earlier identification of high-risk patients, enabling timely and targeted interventions. Future studies are warranted to validate these findings in larger cohorts and explore potential therapeutic strategies aimed at modulating these markers to improve outcomes in ICU settings.

## Figures and Tables

**Table 1:** Sociodemographic and laboratory data of patients in the intensive care unit (ICU).

	Patients (N = 160) Mean ± SD	Normal Range for Healthy Individual
Age (years)	60.5 ± 15.8	
Gender Male Female	92 (58%) 68 (42%)	
Cardiovascular disease, n (%)	72 (45%)	
Respiratory system disease, n (%)	58 (36%)	
Chronic kidney disease (CKD)	38 (24%)	
Diabetes, n (%)	61 (38%)	
Hypertension, n (%)	68 (42%)	
WBC (×10^3^/µL)	11.2 ± 3.8	4.0–11.0
RDW (%)	14.5 ± 2.1	11.5–14.5
Platelet (×10^3^/µL)	190 ± 58	150–450
Neutrophil (×10^3^/µL)	7.5 ± 2.9	1.5–8.0
MPV (fL)	8.9 ± 1.2	7.5–11.5
Red blood cell (RBC; ×10^3^/µL)	4.8 ± 0.5	4.5–5.9 (male) 4.0–5.2 (female)
Hemoglobin (Hb; g/dL)	14.5 ± 1.3	13.8–17.2 (male) 12.1–15.1 (female)
Monocyte (×10^3^/µL)	0.7 ± 0.3	0.2–0.8
Lymphocyte (×10^3^/µL)	1.0 ± 0.4	1.0–4.8
Platelet-to-lymphocyte ratio (PLR)	180 ± 35	100–300
Neutrophil-to-lymphocyte ratio (NLR)	7.1 ± 3.0	1–3
Pan-immune-inflammation value (PIV)	540 ± 110	300–600
Ca^++^ (mg/dL)	8.9 ± 0.7	8.5–10.5
CRP (mg/L)	65 ± 22	<10
Procalcitonin (ng/L)	2.8 ± 1.2	<0.5
Vitamin D (ng/mL)	18.5 ± 6.2	20–50
Gla-rich protein (GRP; μg/mL)	0.81 ± 0.6	0.3–0.8
dp-ucMGP (pmol/L)	720 ± 225	300–800
Hospital stay (days)	15.3 ± 6.5	
Survivor Non-survivor	120 (75%) 40 (25%)	

**Table 2:** Comparison of parameters of survival and mortality of patients in the ICU.

Parameters	Survivor (N = 120) Mean ± SD	Mortality (N = 40) Mean ± SD	*p*-Value
WBC (×10^3^/μL)	10.5 ± 3.2	12.0 ± 3.8	0.05
RDW (%)	14.0 ± 1.8	15.2 ± 2.4	0.03
Platelet (×10^3^/μL)	210 ± 60	160 ± 50	0.01
Neutrophil (×10^3^/μL)	7.0 ± 2.5	8.5 ± 3.0	0.02
MPV (fL)	8.6 ± 1.0	9.2 ± 1.2	0.05
Monocyte (×10^3^/μL)	0.7 ± 0.3	0.9 ± 0.4	0.05
Lymphocyte (×10^3^/μL)	1.2 ± 0.4	0.9 ± 0.3	0.03
Platelet-to-lymphocyte ratio (PLR)	175 ± 35	230 ± 50	0.02
Neutrophil-to-lymphocyte ratio (NLR)	6.0 ± 2.5	9.0 ± 3.5	0.01
Pan-immune-inflammation value (PIV)	450 ± 110	610 ± 120	0.01
Ca^++^ (mg/dL)	8.8 ± 0.6	8.4 ± 0.7	0.04
CRP (mg/L)	32 ± 10	78 ± 25	0.01
Procalcitonin (ng/L)	2.5 ± 1.1	3.8 ± 1.5	0.03
Vitamin D (ng/mL)	19.0 ± 6.0	12.5 ± 5.0	0.02
Gla-rich protein (GRP; ng/mL)	0.76 ± 0.5	0.98 ± 0.6	0.03
dp-ucMGP (pmol/L)	650 ± 210	920 ± 180	0.02

**Table 3:** The correlation between the length of the hospital stay and the mortality of patients in the intensive care unit (ICU).

Variables	Hospital Stay (Days) R Value, *p*-Value	ICU Mortality R Value, *p*-Value
WBC (×10^3^/μL)	0.28, 0.02	0.35, 0.01
RDW (%)	0.30, 0.01	0.40, 0.01
Platelet (×10^3^/μL)	−0.20, 0.04	−0.25, 0.03
Neutrophil (×10^3^/μL)	0.22, 0.03	0.30, 0.02
MPV (fL)	0.18, 0.05	0.25, 0.03
Monocyte (×10^3^/μL)	0.10, 0.10	0.15, 0.08
Lymphocyte (×10^3^/μL)	−0.25, 0.03	−0.30, 0.02
Platelet-to-lymphocyte ratio (PLR)	0.32, 0.01	0.35, 0.01
Neutrophil-to-lymphocyte ratio (NLR)	0.40, 0.01	0.45, 0.01
Pan-immune-inflammation value (PIV)	0.38, 0.01	0.42, 0.01
Ca^++^ (mg/dL)	−0.15, 0.08	−0.18, 0.06
CRP (mg/L)	0.28, 0.02	0.32, 0.01
Procalcitonin (ng/L)	0.35, 0.01	0.40, 0.01
Vitamin D (ng/mL)	−0.30, 0.02	−0.40, 0.01
GRP (ng/mL)	0.35, 0.01	0.38, 0.01
dp-ucMGP (pmol/L)	0.40, 0.01	0.50, 0.001

**Table 4:** Linear regression analysis results for the mortality of patients in the ICU.

Variable	B	St. Error	Beta	t	*p*-Value
WBC (×10^3^/μL)	0.15	0.05	0.20	2.98	0.01
RDW (%)	0.30	0.07	0.35	4.29	0.001
Platelet (×10^3^/μL)	−0.10	0.04	−0.25	−2.50	0.02
Neutrophil (×10^3^/μL)	0.18	0.06	0.22	3.00	0.01
MPV (fL)	0.08	0.03	0.20	2.67	0.02
Monocyte (×10^3^/μL)	0.12	0.05	0.15	2.40	0.03
Lymphocyte (×10^3^/μL)	−0.20	0.06	−0.25	−3.33	0.01
Platelet-to-lymphocyte ratio (PLR)	0.22	0.07	0.30	3.14	0.01
Neutrophil-to-lymphocyte ratio (NLR)	0.35	0.08	0.40	4.38	0.001
Pan-immune-inflammation value (PIV)	0.40	0.09	0.42	4.44	0.001
Ca^++^ (mg/dL)	−0.10	0.04	−0.18	−2.50	0.02
CRP (mg/L)	0.28	0.07	0.32	3.57	0.01
Procalcitonin (ng/L)	0.35	0.08	0.40	4.38	0.001
Vitamin D (ng/mL)	−0.40	0.10	−0.45	−4.00	0.001
GRP (ng/mL)	0.25	0.07	0.35	3.57	0.01
dp-ucMGP (pmol/L)	0.20	0.06	0.30	3.25	0.01

**Table 5:** Multiple logistic regression analysis results for the mortality of patients in the intensive care unit (ICU).

	ICU Mortality		
	Odds Ratio	95% CI	*p*-Value
WBC (×10^3^/μL)	1.20	1.05–1.35	0.02
RDW (%)	1.35	1.10–1.60	0.01
Platelet (×10^3^/μL)	0.85	0.75–0.95	0.02
Neutrophil (×10^3^/μL)	1.25	1.10–1.40	0.01
MPV (fL)	1.20	1.05–1.35	0.02
Monocyte (×10^3^/μL)	1.10	0.95–1.25	0.08
Lymphocyte (×10^3^/μL)	0.85	0.75–0.95	0.03
Platelet-to-lymphocyte ratio (PLR)	1.30	1.10–1.50	0.01
Neutrophil-to-lymphocyte ratio (NLR)	1.40	1.20–1.60	0.001
Pan-immune-inflammation value (PIV)	1.50	1.30–1.70	0.001
Ca^++^ (mg/dL)	0.90	0.80–1.00	0.05
CRP (mg/L)	1.32	1.15–1.50	0.01
Procalcitonin (ng/L)	1.45	1.25–1.65	0.001
Vitamin D (ng/mL)	1.30	1.10–1.52	0.001
GRP (ng/mL)	1.40	1.18–1.62	0.001
dp-ucMGP (pmol/L)	1.30	1.12–1.48	0.001

**Table 6:** ROC analysis results in patients with mortality.

	Cut-Off	Sensitivity	Specificity	AUC (95% CI)	*p*-Value
RDW	>14.8	0.78	0.72	0.77 (0.70–0.83)	0.01
PLR	>200	0.81	0.74	0.79 (0.72–0.85)	0.001
NLR	>7.5	0.84	0.76	0.82 (0.75–0.88)	0.001
PIV	>520	0.85	0.79	0.83 (0.77–0.89)	0.001
CRP	>40	0.80	0.75	0.78 (0.72–0.84)	0.01
Procalcitonin	>3.0	0.82	0.74	0.79 (0.73–0.86)	0.001
Vitamin D	<12	0.75	0.72	0.74 (0.68–0.80)	0.01
GRP	>0.90	0.83	0.77	0.82 (0.75–0.88)	0.001
dp-ucMGP	>720	0.85	0.80	0.83 (0.76–0.89)	0.001

AUC: area under the curve; RDW: red cell distribution width; NLR: neutrophil-to-lymphocyte ratio; PLR: platelet-to-lymphocyte ratio; PIV: pan-immune-inflammation value; GRP: Gla-rich protein; dp-ucMGP: dephosphorylated uncarboxylated matrix Gla protein.

## Data Availability

The original contributions presented in the study are included in the article. Further inquiries can be directed to the corresponding authors.
